# Efficacy of Preemptive Analgesia on Pain Perception After Simple Tooth Extraction: A Prospective Study

**DOI:** 10.7759/cureus.58262

**Published:** 2024-04-14

**Authors:** Roohika Sharma, Melvin George, Murugesan Krishnan

**Affiliations:** 1 Oral and Maxillofacial Surgery, Saveetha Dental College and Hospitals, Saveetha Institute of Medical and Technical Sciences, Saveetha University, Chennai, IND

**Keywords:** innovative technique, novel, dental extraction, pain, etoricoxib, pre-emptive analgesia

## Abstract

Background and objective

This study aims to explore the concept of preemptive analgesia, which is the technique of administration of analgesic agents before the painful stimulus. This bridges the time gap between the onset of action of the analgesic agents and the wear-off of local anesthesia. Existing literature also brings up the concept of central sensitization, which is the hyper-activity of the nervous system in response to a noxious stimulus. Administration of preemptive analgesia prevents central sensitization and hence provides prolonged analgesia to the patient. For the benefit of this study, tab. Etoricoxib 90 mg was used as the analgesic agent.

In addition, this study aims to investigate the effects of the administration of tab. Etoricoxib 90 mg 30 minutes before extraction of a single mandibular third molar on the effects of pain experienced by the patient after tooth extraction as compared to a placebo.

Methodology

This was a double-blinded, prospective, observational study. The pain experienced by 50 participants in each group was measured at 1 hour, 6 hours, 12 hours, and 24 hours postoperatively using a visual analog scale (VAS). The independent samples t-test was then conducted to evaluate the results and draw out conclusions.

Results

The average difference in pain experienced was maximum in the first hour after the procedure. The mean VAS score reported by patients was 3.14 in the study group but was 6.40 in the control group within the first hour. This difference was reduced in the first six hours after the procedure, with the average score being 3.82 in the study and 7.16 in the control group. The difference was the least after 12 hours, with the study group experiencing a VAS score of 4.64 and controls experiencing a VAS score of 6.14. After the first 24 hours, the mean VAS score was 3.80 in the study group and 5.60 in the control group.

Conclusions

Preemptive administration of tab. Etoricoxib 90 mg can reduce postextraction pain in healthy adult patients as compared to placebo tablets, with a maximum difference in pain reduction seen at the end of the first six hours (*P *= 0.012) and the minimum at the end of 12 hours (*P *= 0.0197).

## Introduction

Extraction of teeth is physiologically traumatic and is perceived as a painful ordeal by most patients. While several techniques have been tried to relieve pain after dental treatment, most noninvasive techniques are ineffective [1-3]. Conventionally, clinicians use nonsteroidal anti-inflammatory drugs (NSAIDs) to relieve postoperative pain. When a patient undergoes dental extraction, the body undergoes some biochemical changes. Our body perceives dental extraction as a traumatic injury and releases inflammatory markers around the extraction socket similar to any other wound. In response to these inflammatory signals or tissue damage, phospholipids in cell membranes are broken down to release arachidonic acid, which is then converted into prostaglandin H2 (PGH2) through the action of the cyclooxygenase (COX) enzymes. COX-1 is typically expressed in most tissues and plays a role in normal physiological functions, while COX-2 is induced during inflammation [4]. PGH2 is further converted into specific prostaglandins, such as PGE2 and PGF2α, which carry out the process of inflammation [5,6]. Prostaglandins have pro-inflammatory effects, such as promoting vasodilation, increasing vascular permeability, and sensitizing pain receptors (nociceptors), which contribute to the pain and swelling associated with inflammation [7,8]. 

Acute pain can be effectively treated with substances such as NSAIDs [9], including acetaminophen and opioids. Preemptive analgesia works by preventing sensitization of peripheral and central nerve fibers. By administering tab. Etoricoxib 90 mg half an hour before the procedure, we target the inhibition of the release of prostaglandins before central and peripheral sensitization of nerves, thereby preventing allodynia and hyperalgesia. Although a conventional method of administering NSAIDs after noxious stimulus also works on the same principle, the efficacy is lower as the peripheral and central nerve fibers have already undergone sensitization and have a low pain threshold and increased sensitivity to pain [10]. A quick review of the existing literature gives us several conflicting results regarding the concept of central and peripheral nerve sensitization, with several studies showing results in favor of the concept, and several studies showing no clinical differences [10]. While most clinicians do not follow this clinical practice, this study can be used to provide definitive data regarding the efficacy of preemptive analgesia.

Nonselective NSAIDs are the most used group of analgesics, but their added inhibition of COX-1 increases the risk of gastrointestinal symptoms and hence limits their use [11]. Patients who do not respond well to first-line treatments can benefit from Etoricoxib, a selective COX-2 inhibitor [12]. In different cell assays and whole blood assays, Etoricoxib, a second-generation selective COX-2 inhibitor, has shown to have a hundred times more affinity for COX-2 than for COX-1 and is less active against COX-1 than previous generations of selective COX-2 inhibitors [13]. This medication is also useful for treating pain after a range of procedures, ranging from major surgical procedures such as therapeutic knee arthroscopy and total abdominal hysterectomy, and dental procedures such as periodontal surgery, and various other dental treatments [14-17]. This study explores the use of Etoricoxib as a potential preemptive analgesic and its effects on the postoperative pain experienced by the patients.

This study aimed to investigate the effects of administering tab. Etoricoxib 90 mg 30 minutes before dental extraction on the pain experienced by the patient after tooth extraction compared to a placebo. The objectives of the study were to evaluate the effects of the administration of tab. Etoricoxib 90 mg before the extraction of teeth on the pain experienced by the patient and compare the pain experienced by the patient 1 hour, 6 hours, 12 hours, and 24 hours after the procedure.

## Materials and methods

This study was approved by the Institutional Human Ethics Committee (IHEC) of Saveetha Dental College and Hospitals (IHEC number IHEC/SDC/OMFS-2207/23/252). It was a prospective, double-blinded randomized controlled clinical trial with the cases to control the allocation ratio of 1:1 and was carried out at the Department of Oral and Maxillofacial Surgery at Saveetha Dental College and Hospitals. Based on previous studies evaluating similar characteristics, G Power version 3.1 0. software was used to calculate the sample size for the power of 95% was 100. Patients requiring extraction of a single mandibular third molar with dental caries and chronic irreversible pulpitis were included in the study, and all participants were between 18 and 60 years of age. Patients with impacted teeth associated with periapical abscess formation, active pus discharge, and evidence of pericoronitis were excluded from the study. Patients requiring transalveolar extractions or extractions of multiple teeth; patients younger than 18 years of age; immunocompromised patients, including those with diabetes, hypertension, hepatic or renal disorders, or a history of peptic ulcers; and patients with a history of cardiovascular disorders were also excluded from the study.

Both the study and control groups had 50 participants each. The study was performed in our outpatient clinic in May 2023. The study population, consisting of 100 participants, was enrolled within two weeks. Patients entering the clinic underwent a preliminary examination by a dentist, and a thorough medical history and necessary radiographs were taken. Informed consent was obtained from patients who met the eligibility criteria. The dentist then handed the participants a sealed envelope with a number inside. The numbers were generated and then randomly allocated to the cases or control group using Random Allocation Software (RAS version 3.0). It was a double-blind study, with neither the patient nor the clinician aware of the group to which the patient was being allocated.

The study group received a single oral dose of the tab. Etoricoxib 90 mg (brand name - Nucoxia 90) 30 minutes before the procedure, given to the patient by a dental assistant, and the control group received a single oral tablet of a placebo drug, Microcrystalline Cellulose tablets - 250 mg (brand name Zeebo Relief) 30 minutes before the procedure, given to the patient by a dental assistant. After 30 minutes, the patient underwent dental extraction under local anesthesia (2% Lidocaine, 1:80,000 adrenaline) performed by a trained dental surgeon. Post-extraction, patients were prescribed paracetamol 650 mg eighth hourly for three days. One hour post-extraction, patients were asked to rate their pain experience on the visual analog scale (VAS), which ranged from 0 (no pain) to 10 (extreme pain). Further VAS readings were taken at six hours, 12 hours, and 24 hours by telephonic interviews. The VAS is a validated tool for measuring pain intensity, with scores ranging from 0 to 10, where 0 represents no pain and 10 represents the worst pain imaginable. Participants were instructed to mark their pain intensity on a 10 cm horizontal line corresponding to their perceived level of pain.

Statistical analysis

The pain experienced by the patient was recorded on the VAS scale. IBM SPSS Statistics for Windows, Version 23.0 (IBM Corp., Armonk, NY) was used to analyze the collected data. The mean VAS scores at each time point between the two groups were compared using an independent t-test. The level of significance was set as *P* < 0.05.

## Results

A total of 100 participants were included in the study, with 50 participants in each group. The mean age of the patients in the study group was 31.5 years, with ages ranging between 25 and 37 years, while in the control group, the mean age was 36.5 years, with ages ranging between 28 and 44 years. Among the participants, 27 (54%) were male and 23 (46%) were female in the study group and 18 (36%) were male and 32 (64) were female in the control group (Table 1).

**Table 1:** Descriptive characteristics of cases and controls.

		Study group	Control group
Gender	Male	27 (54%)	18 (36%)
	Female	23 (46%)	32 (64%)
Age (in years)	Mean	31.5	36.5
	Range	25-37	28-44

Descriptive analysis

The descriptive characteristics are given in Table 1.

Pain intensity

The results revealed that the study group consistently reported lower pain scores compared to the control group at all time points. The mean VAS scores were significantly lower in the study group compared to the control group at 1 hour (*P* = 0.012), 6 hours (*P* = 0.0172), 12 hours (*P* = 0.0197), and 24 hours (*P* = 0.0202) post-extraction. The mean VAS scores obtained with the standard deviation are depicted in Table 2.

**Table 2:** Mean pain scores among the study participants at different timelines. ^*^Statistically significant - independent sample t-test. SD, standard deviation; VAS, visual analog scale

Time	Group	Sample size	Mean VAS score	SD	Difference in mean VAS score	t	P-value
1 hour	Study group	50	3.14	1.629	3.26	-4.515	0.012*
	Control group	50	6.4	1.309			
6 hours	Study group	50	3.82	1.24	3.34	-5.791	0.0172*
	Control group	50	7.16	1.218			
12 hours	Study group	50	4.64	1.396	1.50	-2.415	0.0197*
	Control group	50	6.14	1.69			
24 hours	Study group	50	3.8	1.429	1.80	-2.554	0.0202*
	Control group	50	5.6	2.04			

## Discussion

The extraction of teeth is a physiologically traumatic process that induces several biochemical reactions in the body. One of these reactions includes the activation of the pain pathway and sensitization of central and peripheral neurons, which causes severe pain that is experienced by the patients up to a week after the procedure. While some measures have proven to be successful, such as administration of corticosteroids, more extensive and noninvasive methods of analgesia are still required. Several patients have reported continuous pain even before the effects of local anesthesia wear off, hence proving the need for better analgesics [18-22].

Preemptive analgesia refers to the concept of administration of analgesics before the onset of the painful stimulus, which can provide long-term pain relief to the patient. This hypothesis is in accordance with similar studies done before, such as those done by Steffens et al., who hypothesized that the preemptive use of 120 mg of Etoricoxib can relieve the postoperative pain after periodontal open-flap debridement surgery [23,24].

The findings of this study provide evidence supporting the efficacy of preemptive analgesia using tab. Etoricoxib 90 mg in reducing postoperative pain following tooth extractions. The lower VAS scores observed in the experimental group indicate that administering Etoricoxib 30 minutes before the procedure has a beneficial effect on pain management, at least within the first 24 hours postoperatively. These results can be explained by the prevention of central sensitization. The hypothesis is that preemptive use of analgesics prevents the release of prostaglandins and hence prevents the lowering of the firing threshold for the surrounding nociceptors. This, in turn, reduces the activation threshold of nociceptors, which prevents pain experience well after the effects of local anesthesia have worn off.

It can also be said that the reduced pain is due to the bridging of the time period between the wear-off of local anesthesia and the onset of action of Etoricoxib. While this would explain the reduced VAS score in the first hour after extraction, it cannot explain the prolonged difference apparent in the first 24 hours. This can only be explained by the prevention of activation of the pain pathway by preemptive analgesia. In our study, a per-oral dose of Etoricoxib 90 mg prevented pain better than a placebo for the first 24 hours following dental extraction, with the difference in the pain experience being maximum at the 24-hour mark and minimum at the 12-hour period.

A similar study done by Gupta et al. proved that a dose of Etoricoxib 60 mg was found to be quite efficient at reducing discomfort during fixed orthodontic appliance therapy [25]. Studies show that postoperative analgesia with Etoricoxib can lessen nervous stimulation in a model of acute pain [26]. Our study is comparable to the ones stated previously. Costa et al. found that a preemptive tablet of 120 mg Etoricoxib significantly reduced pain as compared to placebo, within the first 48 hours following surgery with no adverse effects [27]. Some drawbacks of Costa et al.'s study include a small sample size, which can reduce the probability of adverse effects.

Studies by Malmstrom et al. and Albuquerque et al. also concluded that during the 24 hours of the study period, 52% of patients in the group administered with Etoricoxib (90 mg) and 81.6% in the group given placebo medication required additional analgesics [28], hence proving that an increase in the pain threshold can be seen with administration of Etoricoxib before the procedure.

This study demonstrates the efficacy of preemptive analgesia and shows that the implementation of preemptive analgesia protocols in dental practice can have significant clinical implications. By administering a single dose of Etoricoxib 90 mg 30 minutes before tooth extraction, clinicians can achieve superior pain control and improve patient satisfaction.

Limitations of the study and future directions

This study focused exclusively on the short-term effects of preemptive analgesia with Etoricoxib up to 24 hours post-extraction. Therefore, the long-term duration of analgesia provided by Etoricoxib and its sustainability beyond 24 hours remain unknown. Future studies should investigate the extended analgesic effects of Etoricoxib and explore its impact on the overall healing process.

## Conclusions

In conclusion, the administration of tab. Etoricoxib 90 mg 30 minutes before dental extraction significantly reduces postoperative pain experienced by the patient as compared to a placebo. The difference in the pain experienced is maximum in the first 6 hours and continues till 24 hours after the procedure. By expanding our understanding of preemptive analgesia, dental professionals can optimize pain management strategies and improve patient outcomes in routine dental practice.
